# Influence of capillary tube length on the performance of domestic refrigerator with eco-friendly refrigerant R152a

**DOI:** 10.1038/s41598-022-18606-w

**Published:** 2022-08-24

**Authors:** A. Baskaran, N. Manikandan, LetaTesfaye Jule, N. Nagaprasad, Abel Saka, Bayissa Badassa, Krishnaraj Ramaswamy, Venkatesh Seenivasan

**Affiliations:** 1 Department of Mechanical Engineering, P.A. College of Engineering and Technology, Pollachi, Tamil Nadu 642002 India; 2 Department of Physics, College of Natural and Computational Science, Dambi Dollo University, Dembi Dolo, Ethiopia; 3 Centre for Excellence-Indigenous Knowledge, Innovative Technology Transfer and Entrepreneurship, Dambi Dollo University, Dembi Dolo, Ethiopia; 4 Department of Mechanical Engineering, ULTRA College of Engineering and Technology, Madurai, Tamilnadu 625104 India; 5 Ministry of Innovation and Technology, Addis Ababa, Ethiopia; 6 Department of Mechanical Engineering, Dambi Dollo University, Dembi Dolo, Ethiopia; 7 Department of Mechanical Engineering, Sri Eshwar College of Engineering, Coimbatore, India

**Keywords:** Environmental sciences, Energy science and technology, Engineering, Materials science

## Abstract

The household heating and cooling system often use the capillary device. The use of the helical capillary eliminates the need for lightweight refrigeration devices in the system. Capillary pressure is noticeably affected by the capillary geometric parameters, such as length, mean diameter, and pitch. This paper is concerned with the effects of the capillary length on the performance of the system. Three separate length capillary tubes were used in the experiment. The data on R152a were studied under various conditions to assess the impact of varying the length. Maximum COP is obtained at an evaporator temperature of − 12 °C and capillary length of 3.65 m. The result is drawn that the system performance enhances when the capillary length is improved to 3.65 m when compared to 3.35 m and 3.96 m. As a result, as the capillary length increases up to a specific amount, the system's performance improves. The findings from the experiment were compared with those from the computational fluid dynamics (CFD) analysis.

## Introduction

A refrigerator is a cooling appliance comprising a thermally insulated compartment, and a refrigeration system is a system that produces a cooling effect in the insulated compartment.As refrigeration is defined as a process of removing heat from a space or substance and transferring that heat to another space or substance. Nowadays, refrigerators are extensively used to store foods which deteriorate at ambient temperatures; spoilage from bacterial growth and other processes is much slower in the refrigerator that has low temperatures. The refrigerant is the working fluid used as a heat absorber or cooling agent in the refrigeration process. The refrigerant collects heat by evaporating at low temperatures and pressures and then condenses at higher temperatures and pressures to release it. The region appears to cool as the heat is evacuated from the refrigerated chamber. The refrigeration process takes place in a system that includes a compressor, a condenser, a capillary, and an evaporator. The refrigerator is the refrigeration plant employed in this study. Refrigerators are widely used around the world, and this equipment has become a home need. The performance of a modern refrigerator is quite efficient, but research to improve the system is still underway. One main disadvantage of R134a is that it is known to be non-toxic but has a very high Global Warming Potential (GWP). R134a, used in domestic refrigerators, was incorporated in the Kyoto Protocol of the United Nations Framework Convention on Climate Change^1,2^. As a consequence, however, R134a use must be significantly decreased^3^. From the ecological, fiscal, and health problems, it is important to find low global warming refrigerants^4^. Multiple researches have proven that R152a is an ecologically sustainable refrigerant. Mohanraj et al.^5^ investigated the theoretical feasibility of employing R152a and hydrocarbon refrigerants in household refrigerators. Hydrocarbons have been found to be inefficient as standalone refrigerants. R152a is more energy-efficient and environmentally friendly than phase-out refrigerants. Bolaji et al.^6^. In a vapour compression refrigerator, the performance of three eco-friendly HFC refrigerants was compared. They came to the conclusion that R152a can be employed in vapour compression systems and can be substituted for R134a. R32 has disadvantages such as high pressure and a low coefficient of performance (COP). Bolaji et al.^7^ tested R152a and R32 as replacements for R134a in a residential refrigerator. According to the research, R152a has a 4.7 percent higher average COP than R134a.R152a and R134a were tested in a refrigeration plant with a hermetic compressor by Cabello et al. ^8^. R152a was tested in a refrigeration system by Bolaji et al.^9^. They concluded that the R152a was the most energy-efficient, with refrigeration power per tonne of 10.6% less than that of the previous R134a. Higher Volumetric refrigeration Capacity and COP were demonstrated in R152a. Chavhan et al.^10^ analyzed the performance of R134a and R152a. In the research of two refrigerants investigated, R152a was shown to be the most energy-efficient. R152a has a COP of 3.769 percent greater than R134a and can be used as a drop-in substitute. Bolaji et al.^11^ looked studied various low GWP refrigerants as alternatives to R134a in refrigeration systems because they have a low global warming potential. The highest energy performance of the refrigerants evaluated was R152a, which used 30.5 percent less electricity per tonne of refrigeration than R134a. R161 will need to be redesigned completely before it can be used as a replacement, according to the authors. Many researchers conducted various experimental works in the domestic refrigerator to enhance the performance of the system with low GWP refrigerants and blend with R134a as a forthcoming alternative replacement in the refrigeration system^12–23^. Baskaran et al.^24–35^ examined the performance of several eco-friendly refrigerants and combinations with R134a as a prospective alternative replacement in various tests on a vapour compression refrigeration system. Tiwari et al.^36^ used experimental and CFD analysis to compare the performance of capillary tubes with different refrigerants and tube diameters. The analysis is carried out using the ANSYS CFX software. The best helical coiled design is recommended. Punia et al.^16^ investigated the effect of capillary tube length, diameter, and coil diameter on LPG refrigerant mass flow rate through helical coil tubes. Adjusting the capillary length range between 4.5 and 2.5 m boosted the mass flow rate by an average of 25%, according to the findings. Söylemez et al.^16^ used three different turbulence (viscous) models to perform a CFD analysis for a fresh food compartment of a domestic refrigerator (DR) to gain insight into not only the cooling time rate of the fresh food compartment but also the air and temperature distribution inside the compartment when it was loaded. The predictions of the developed CFD model vividly illustrate the airflow and temperature fields inside the FFC.

This paper examines the findings of an experimental investigation conducted to establish the performance of a residential refrigerator using R152a refrigerant, which is environmentally beneficial and has no risk for ozone depression potential (ODP).

In this research, the capillary tube lengths of 3.35 m, 3.65 m, and 3.96 m are selected as test sections. Then experiments were conducted with the low global warming refrigerant R152a, and the performance parameters were calculated. The refrigerant behaviour in the capillary tube is also analyzed using the CFD software. The results of the CFD were compared to the results of the experiments.

## Materials and methods

### Setup for experiments

**Figure 1:**
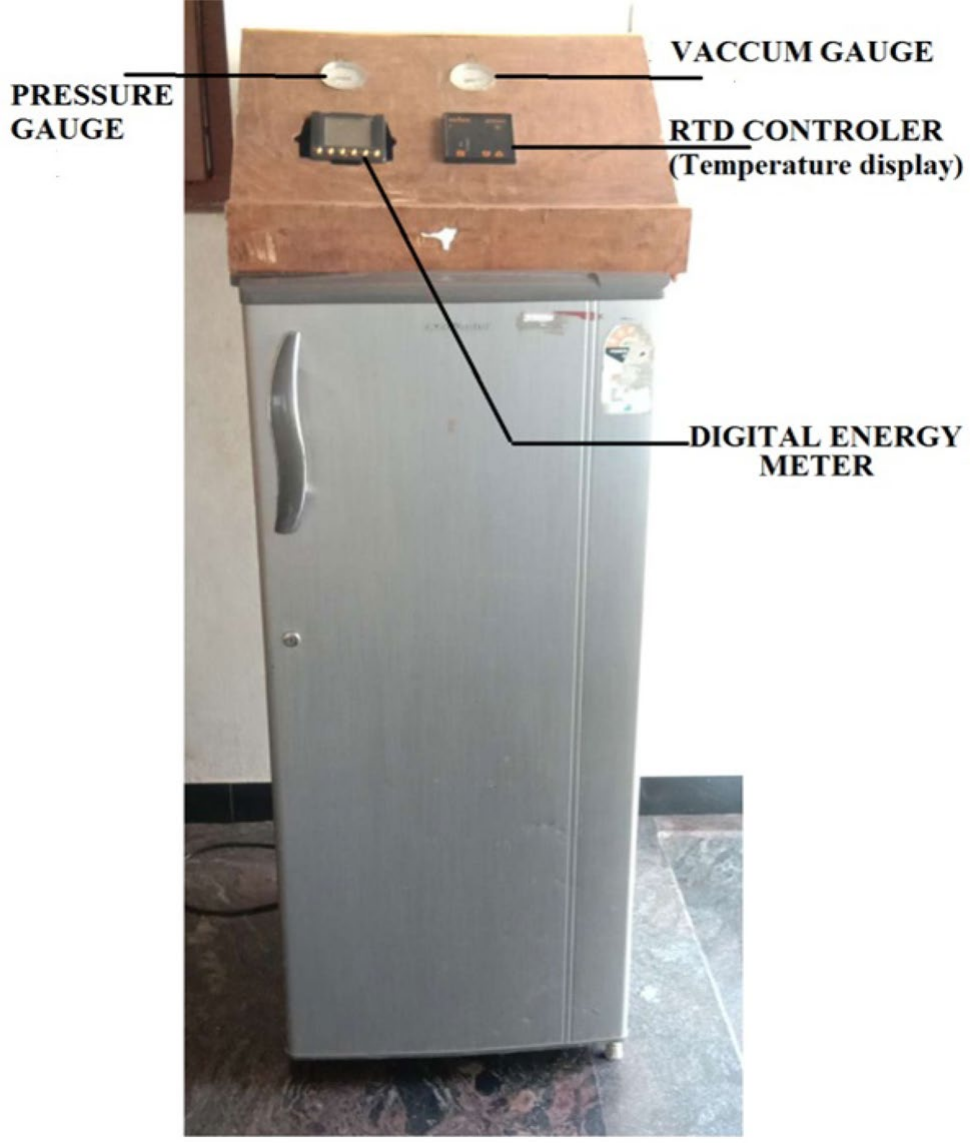
View of the experimental set.

As shown in Fig. 1, a photographic representation of a 185-L domestic fridge, which is intended for research, is seen. It consists of an evaporator, a hermetically sealed reciprocating compressor, and an air-cooled condenser. At the compressor intake, condenser inlet, and evaporator outlet, there were four gauges. To prevent vibration during testing, these gauges were installed on a panel. To read the thermocouple temperature, all of the thermocouple wires are connected to the thermocouple scanner. Ten temperature measures were mounted at the evaporator inlet, compressor suction, compressor discharge, the refrigerator compartment and inlet, condenser inlet, freezer, and the condenser outlet. The consumed voltage and current were also reported as well. The flow measuring instrument which was attached to the piping link was fixed to the wooden panel. Records were stored every 10 s using a Human Machine Interface (HMI) unit. A sight glass is used to check the uniformity of a condensed liquid's flow.

To quantify the power and energy, a Selec MFM384 energy metre with a 100–500 V input voltage was used. System service ports were installed on the top of the compressor for charging and recharging the refrigerant. The first step was to drain the system of moisture via service ports. To clear the system of any pollutants, it was purged with nitrogen gas. The system was charged using a vacuum pump, which evacuated the device to a pressure of − 30 mm of mercury. Table 1 shows the technical specifications of the domestic refrigerator test unit, whereas Table 2 shows the measured quantities, as well as their range and precision.

**Table 1:** Technical parameters of a Household refrigerator.

Volume of storage	169 L
Range of current	1.10 max
Range of volt	220–240 V
Range of frequency	50 Hz
Doors	1
Type of refrigerant	R134a
Method of defrost system	Auto defrost
Amount of charge	140 Grams
length of capillary tube	3.35 m
Inner diameter of capillary tube	0.00078 m
Capacity of cooling	182 Watts

**Table 2:** Items measured, together with their range and precision.

Items	Range	Precision
Temperature	− 40 °C to 110 °C	+ 0.1 °C
Power consumption	0–1000 W	1 W
Voltage	0–240 V	0.1 V
Current	0–10 A	0.1 A
Pressure	0–150 MPa	+ 0.7 kPa
Refrigerant flow meter	0–100 cc/s	0.1 cc/s

The characteristics of refrigerants used in domestic refrigerators and freezers are shown in Table 3.

**Table 3:** The characteristics of refrigerants.

Refrigerant	Molar mass (kg/kmol)	Boiling point (℃)	Critical temp (℃)	Critical pressure (Mpa)	Critical density (kg/m^3^)	Latent heat (KJ/kg)	ODP	GWP
R134a	102.03	− 26.074	101.06	4.059	511.9	216.7	0	1370
R152a	66.051	− 24.023	113.26	4.516	368.0	329.5	0	133

## Test procedure

The tests were carried out in accordance with the ASHRAE handbook 2010 recommendations, under the following conditions:

- Freezer Unit: − 19 to − 16 °C
- Perishable Unit: 4–6 °C
- Ambient temperature: 26–33 °C.

For good measure, furthermore, checks were performed to ensure the repeatability of the results. Temperature, pressure, refrigerant flow rate, and energy consumption were collected whilst the working conditions were kept in a stable state. Temperature, pressure, energy, power, and flow rate were all measured to determine the system's performance characteristics. Using a given temperature, the refrigeration effect and COP are found for specific values of mass flow rate and power.

## CFD analysis

### Pre-processing

The influence of capillary length can be easily calculated by utilizing CFD analysis for the dual-phase flows inside helically coiled tubes in domestic refrigerators. The CFD analysis makes it simple to track the motion of the fluid particles. Using CFD programme FLUENT, the analysis of refrigerant that passes through inside the helical coil is performed. The dimensions of the capillary coils are presented in Table 4.

**Table 4:** Geometrical parameters of capillary coils.

Parameters	Coil1	Coil2	Coil3
Coil inner diameter (mm)	0.7874	0.7874	0.7874
Pitch (mm)	5	5	5
Coil mean diameter (mm)	50.4	50.4	50.4
Number of turns	21	23	25
Capillary tube length (mm)	3352.8	3657.6	3962.4

**Figure 2:**
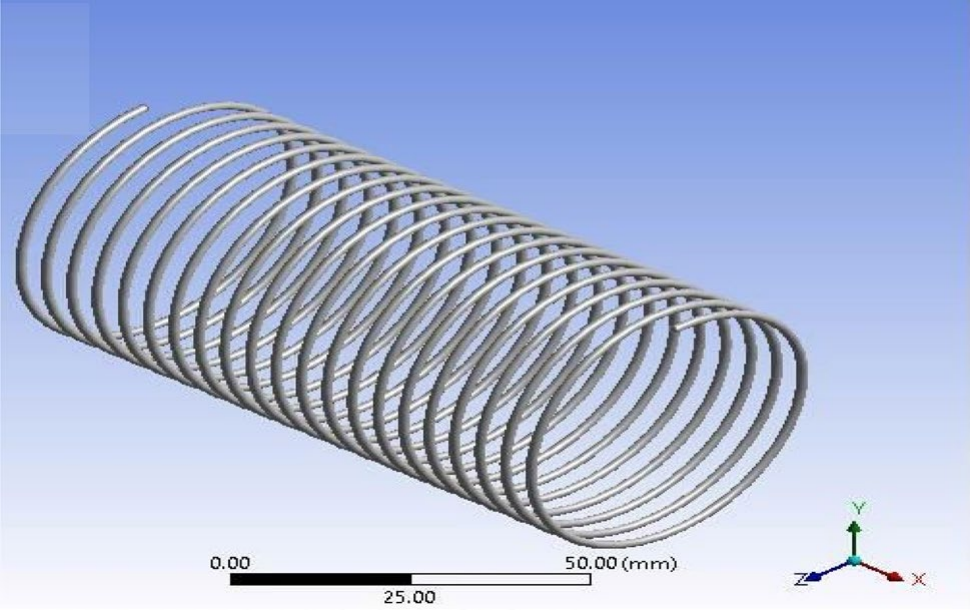
ANSYS FLUENT model of Tube-1 (3352.8 mm).

**Figure 3:**
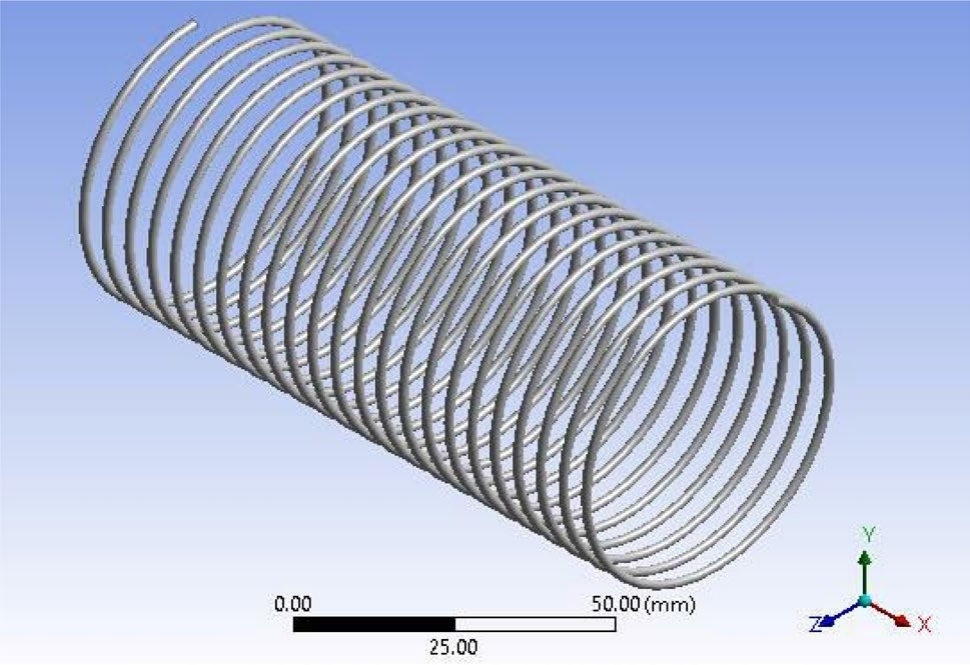
ANSYS FLUENT model of Tube-2 (3657.6 mm).

**Figure 4:**
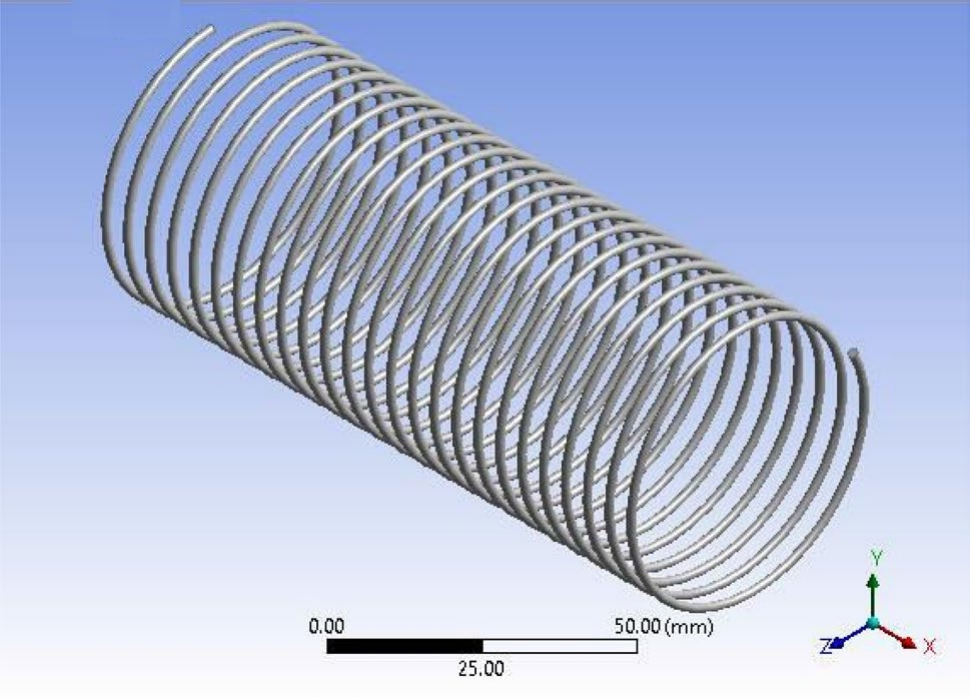
ANSYS FLUENT model of Tube-3 (3962.4 mm).

The mesh modeller of the FLUENT software will produce the design structural model and mesh (Figs. 2, 3 and 4 display the ANSYS Fluent versions.). The pipe fluid volume was used to create the boundary mesh. Here is the grid that was used for this investigation.

The CFD model was developed using the ANSYS FLUENT platform. Only the moving fluid universes were to be represented, so the flow for each capillary coil was modelled based on the capillary's diameter.

**Figure 5:**
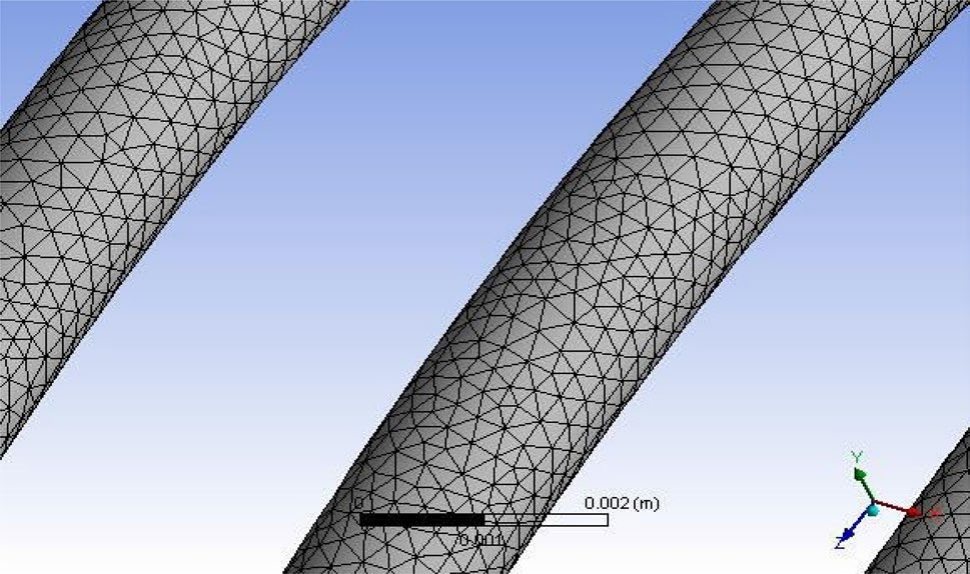
Tetrahedron mesh.

The GEOMETRY model was imported into the ANSYS MESH programme. The ANSYS is working on programme code, in which ANSYS was the model combined and boundary conditions added. Figure 4 shows the ANSYS FLUENT Model of Tube-3 (3962.4 mm). More consistency is provided by the tetrahedron element, which is shown in Fig. 5. After a master mesh was created, the file was saved as a mesh. The lateral face of the coil is known as the inlet, and the opposite side facing the in the outflow. These circular faces remain as pipe walls. Fluid media is used to construct the model.

## Processing

**Figure 6:**
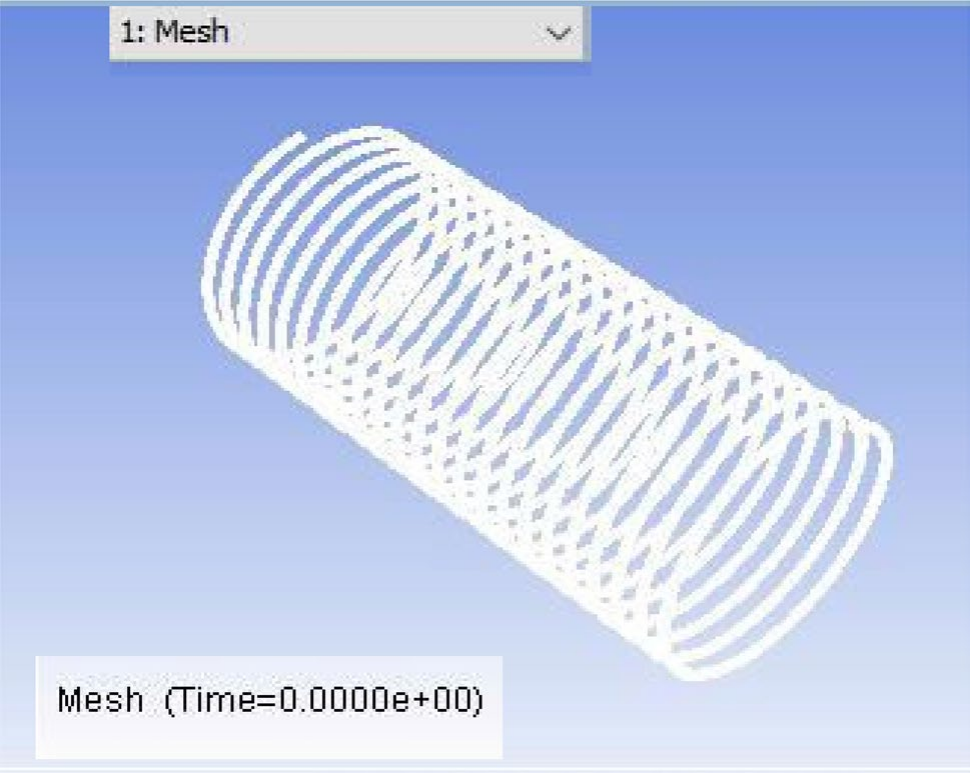
The fluent grid model.

**Figure 7:**
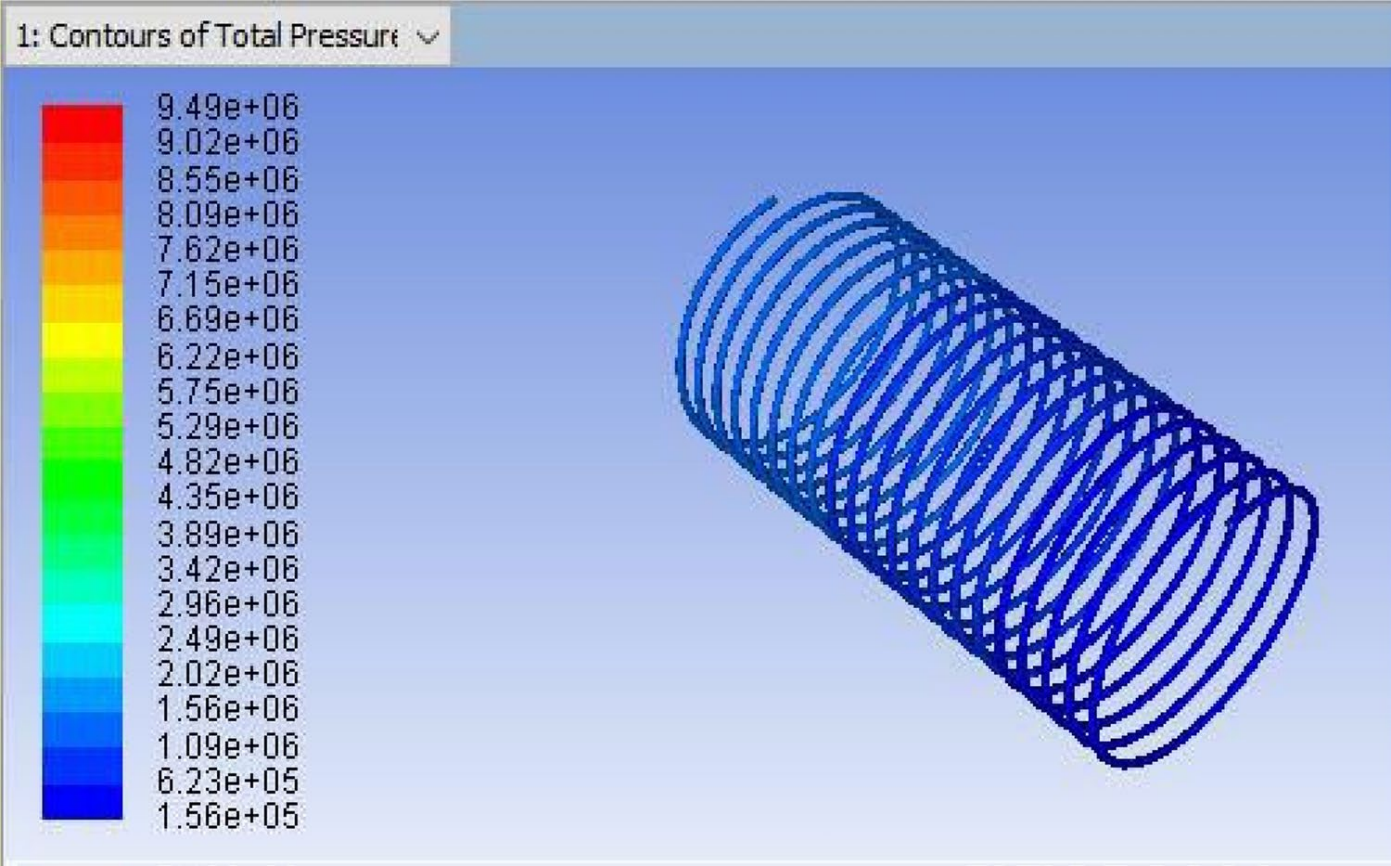
CFD result for a total pressure of Coil-1 (3352.8 mm).

- The program used to find the solution is FLUENT. This will include the following steps:
- The model is converted to millimetres. The solution of the model's grid was tested.
- Pressure and length units are converted to Pascal and millimetres.
- The grids and boundary conditions were visually verified by using the Grid Display option.
- The solution was chosen regardless of how the user felt about the amount of pressure, and the 3D options were selected. Formulas for generating electricity have been turned on.
- When the flow is considered chaotic, it is highly non-linear. In order to meet the K-epsilon flow was thus chosen.
- If a user-specified alternative is chosen, the medium will be the following: The thermodynamic properties of the refrigerant R152a were described. The form properties are stored as database entities.
- Copper was described as the fluid tube wall of the medium.
- The weather patterns were left unchanged. The speed of the inlet, 12.5 bar pressure, was established, and 45 °C was described.
- The outlet was said to be identified by the outflow.
- The answer is left with their default settings.
- The inlet solution was used as the starting point for the solution's calculation.
- Tetrahedron mesh and the Fluent Grid model are depicted in Figs. 5 and 6, respectively.
- Finally, in the fifteenth iteration, the solution was tested, and it converged in the fifteenth iteration, as shown in Fig. 7.

## Post-processing

**Figure 8:**
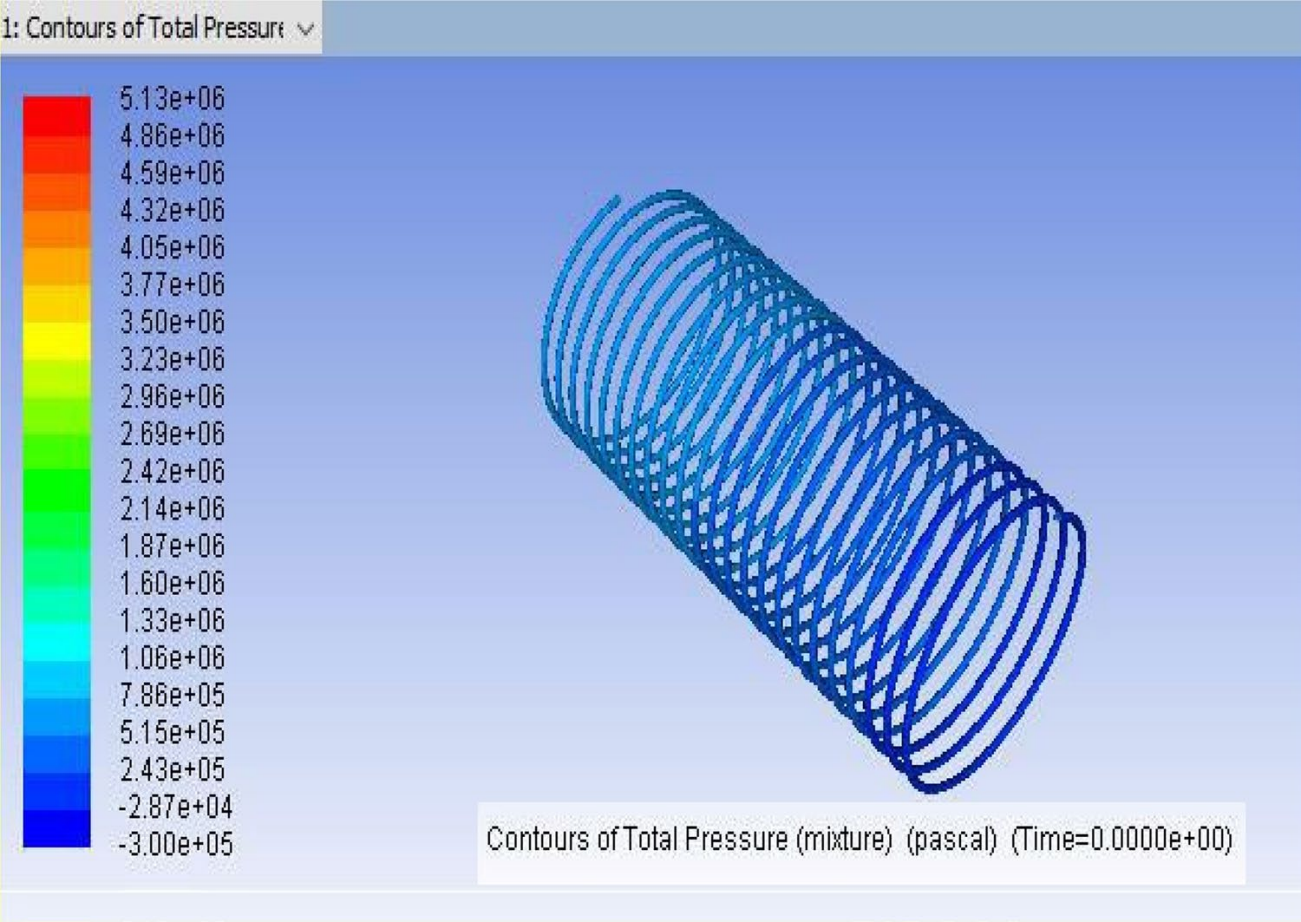
CFD result for a total pressure of Coil-2 (3657.6 mm).

**Figure 9:**
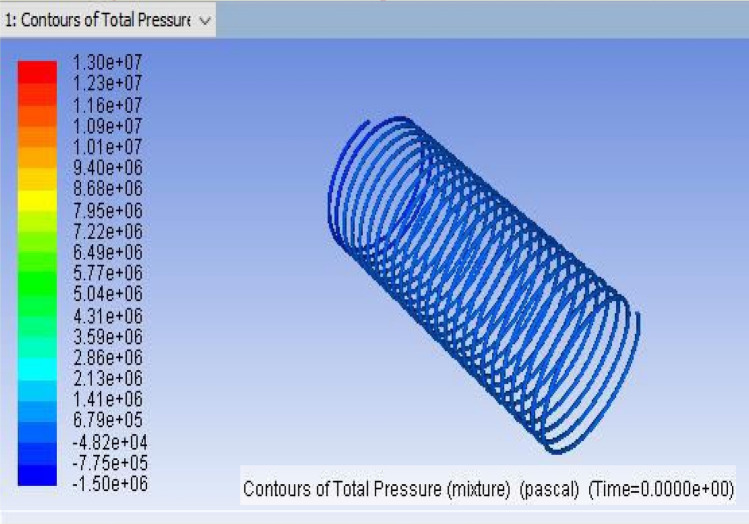
CFD result for a total pressure of Coil-3 (3962.4 mm).

It is the method of mapping and analyzing the findings. The contours of the pressure and temperature data are plotted using the monitor. After this, the overall pressure and temperature and total temperature parameters were determined. This data displays the overall pressure drop for the coils (1, 2 and 3) in Figs. 7, 8 and 9, respectively. These findings were extracted from the fluent programme.

## Result and discussions

**Figure 10:**
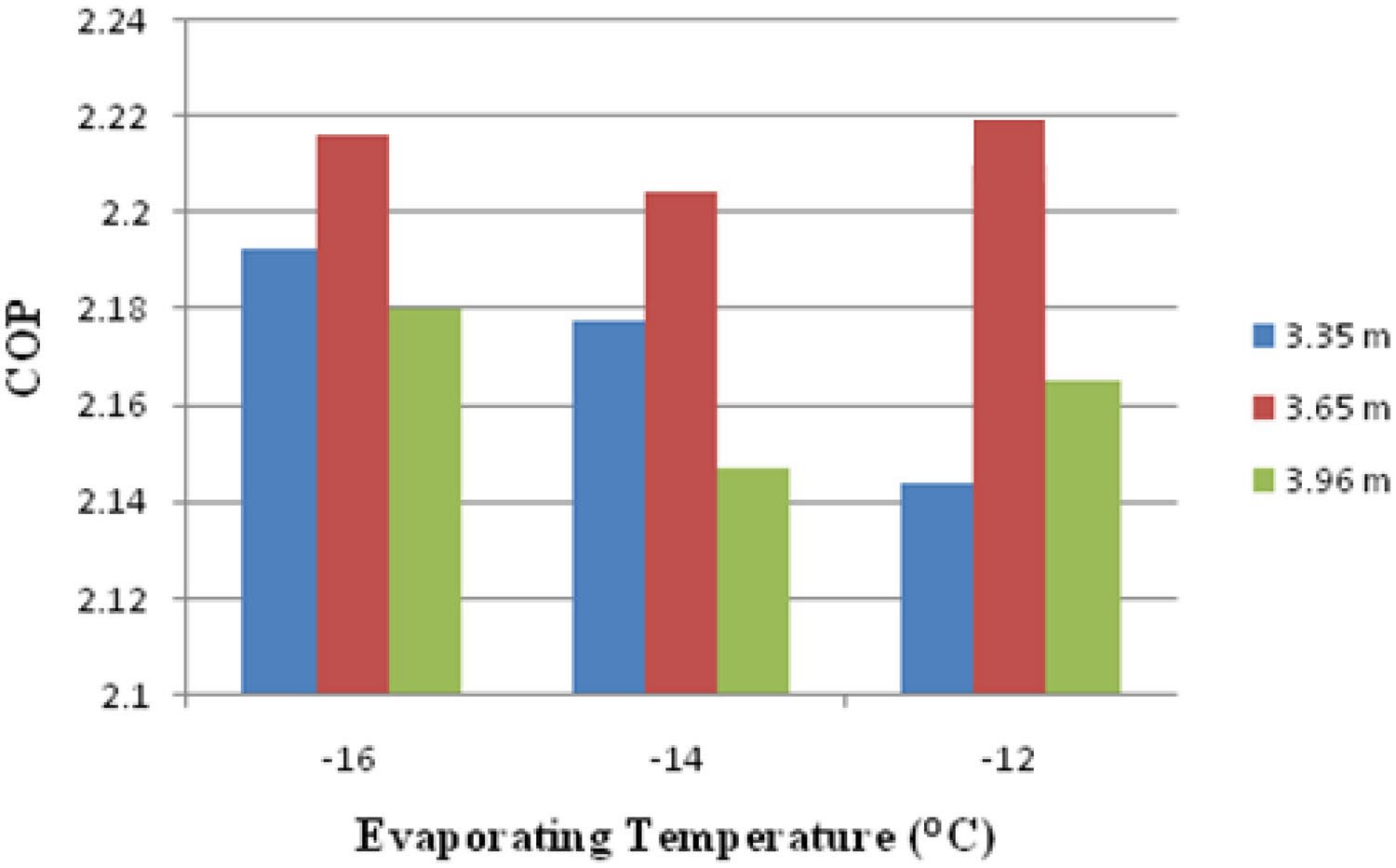
Variation of COP against capillary length and evaporating temperature.

Figure 10 shows the variation of COP vs various evaporation and capillary lengths. The COP increases as the evaporating temperature rise, as shown in the graph. When one gets to the 3.65 m and 3.96 m capillary spans, the most and least COP acquire. If the capillary length increases to a certain amount, the COP decreases.

**Figure 11:**
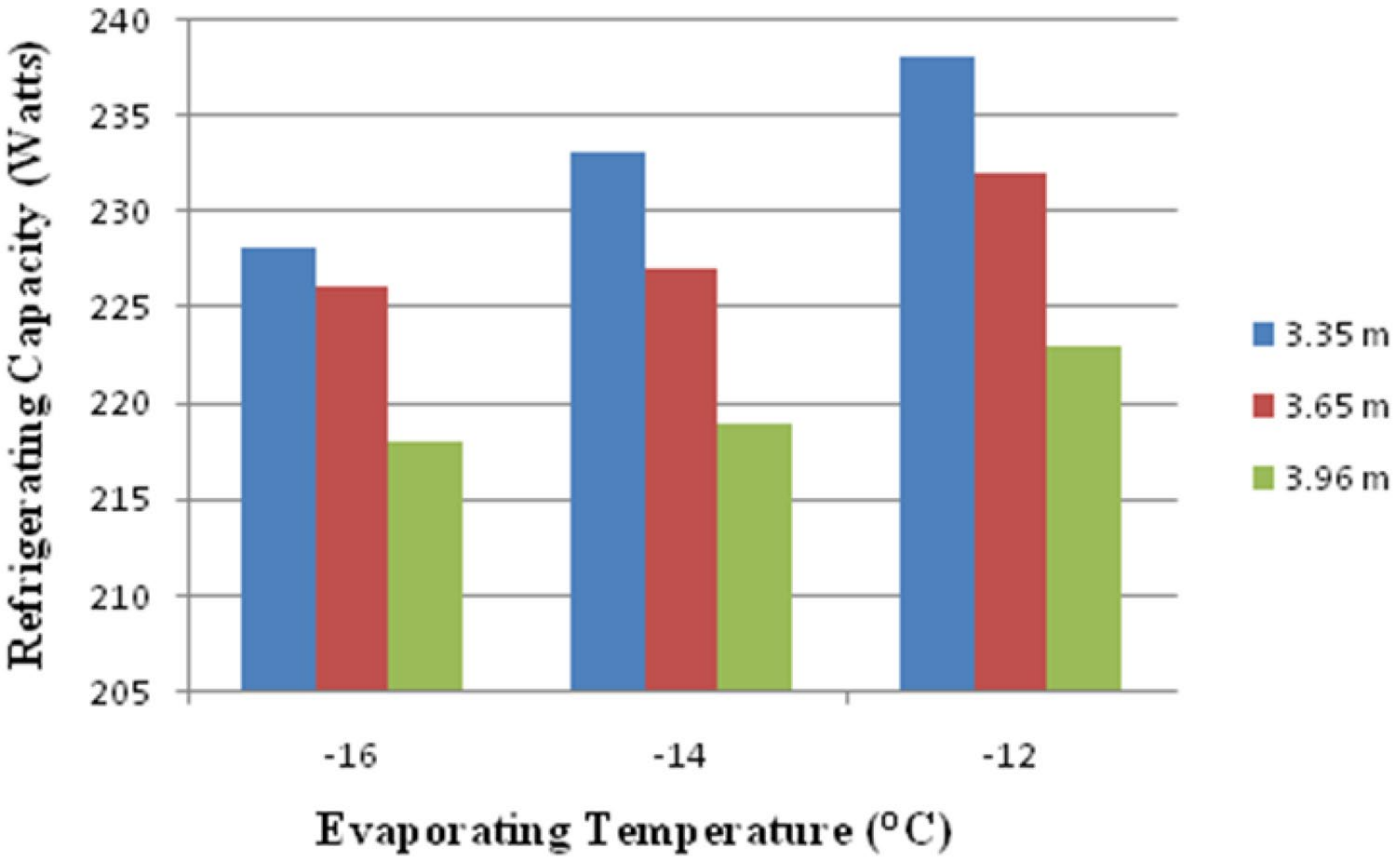
Variation of refrigerating capacity against capillary length and evaporating temperature.

Due to different levels of evaporating temperature and capillary lengths, the variation in Refrigeration capacity is represented in Fig. 11. The Capillary Effect causes a reduction in the Refrigeration capacity. The lowest Refrigeration capacity is obtained at − 16 °C boiling point. The most Refrigeration capacity is observed in the capillary, whose length is about 3.65 m and − 12 °C.

**Figure 12:**
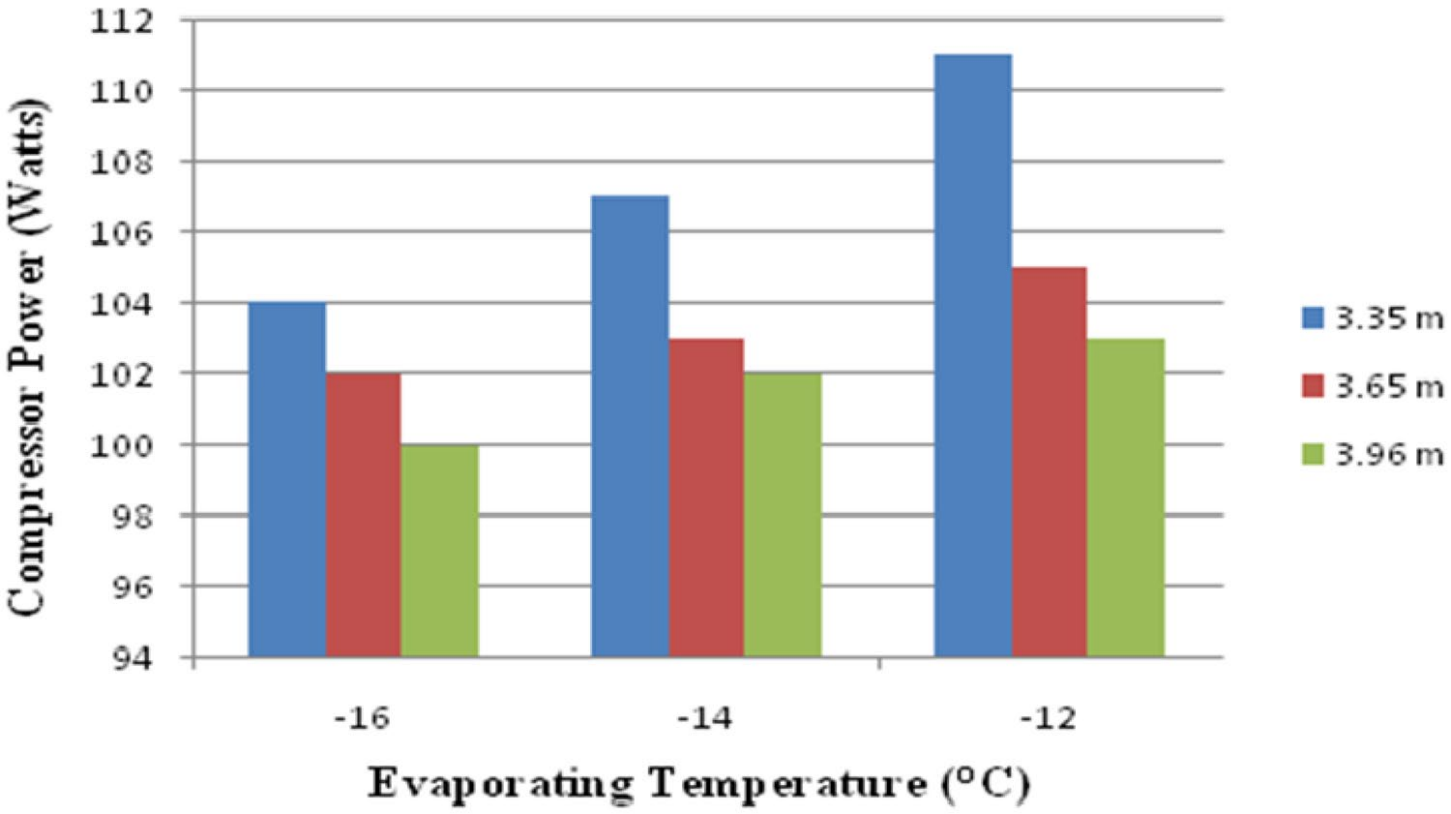
Variation of compressor power against capillary length and evaporating temperature.

Figure 12 shows the variation of compressor power against Capillary Length and evaporating temperature. Furthermore, the graph shows that as the capillary length increases and the evaporation temperature lowers, the power decreases. Lower compressor power is obtained in the capillary length from 3.96 m at − 16 °C evaporating temperature.

## Computational fluid dynamics results

**Figure 13:**
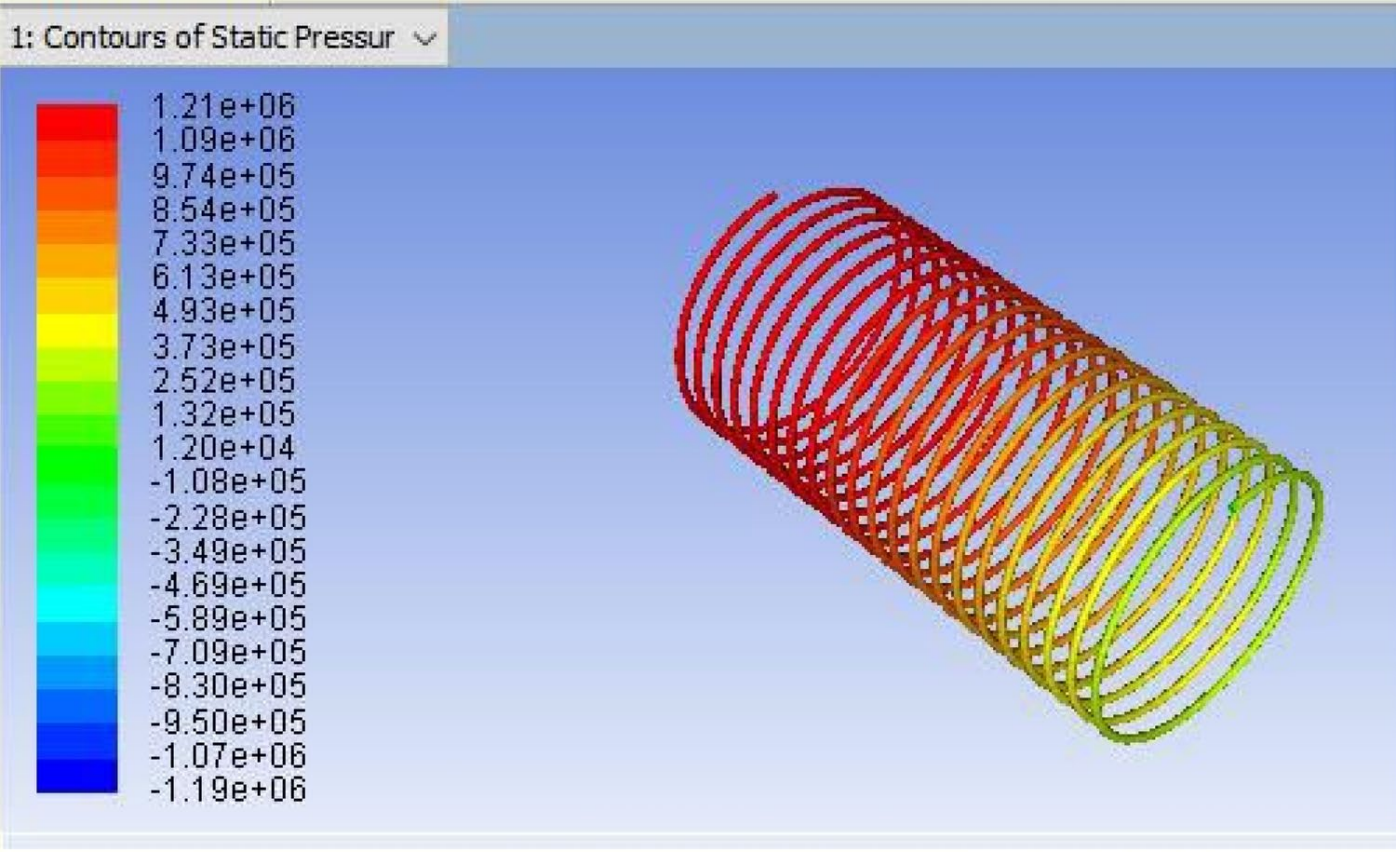
ANSYS Fluent solution of static pressure for capillary coil 1 (3.35 m).

**Figure 14:**
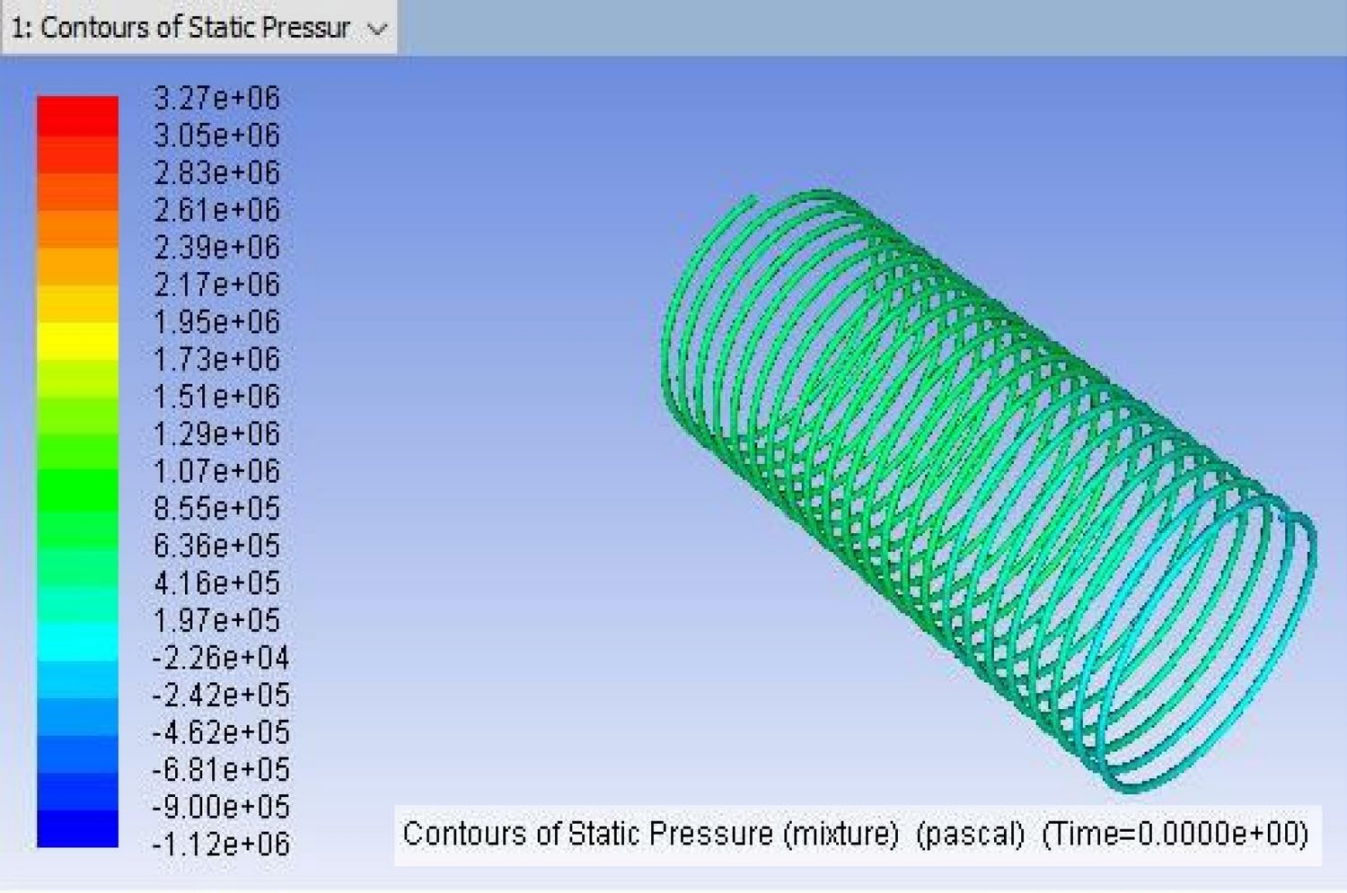
ANSYS Fluent solution of static pressure for capillary coil 2 (3.65 m).

**Figure 15:**
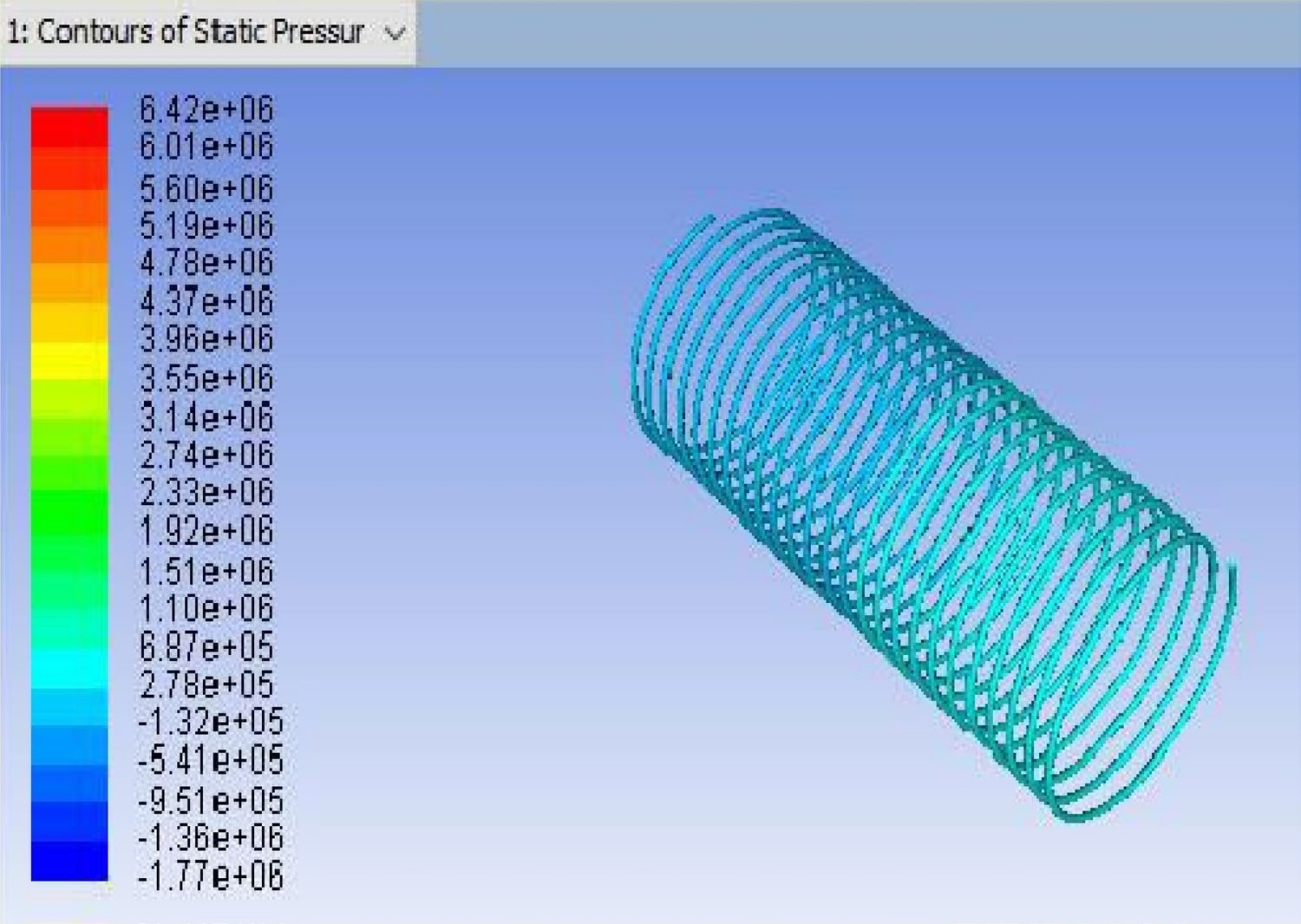
ANSYS Fluent solution of static pressure for capillary coil 3 (3.96 m).

**Figure 16:**
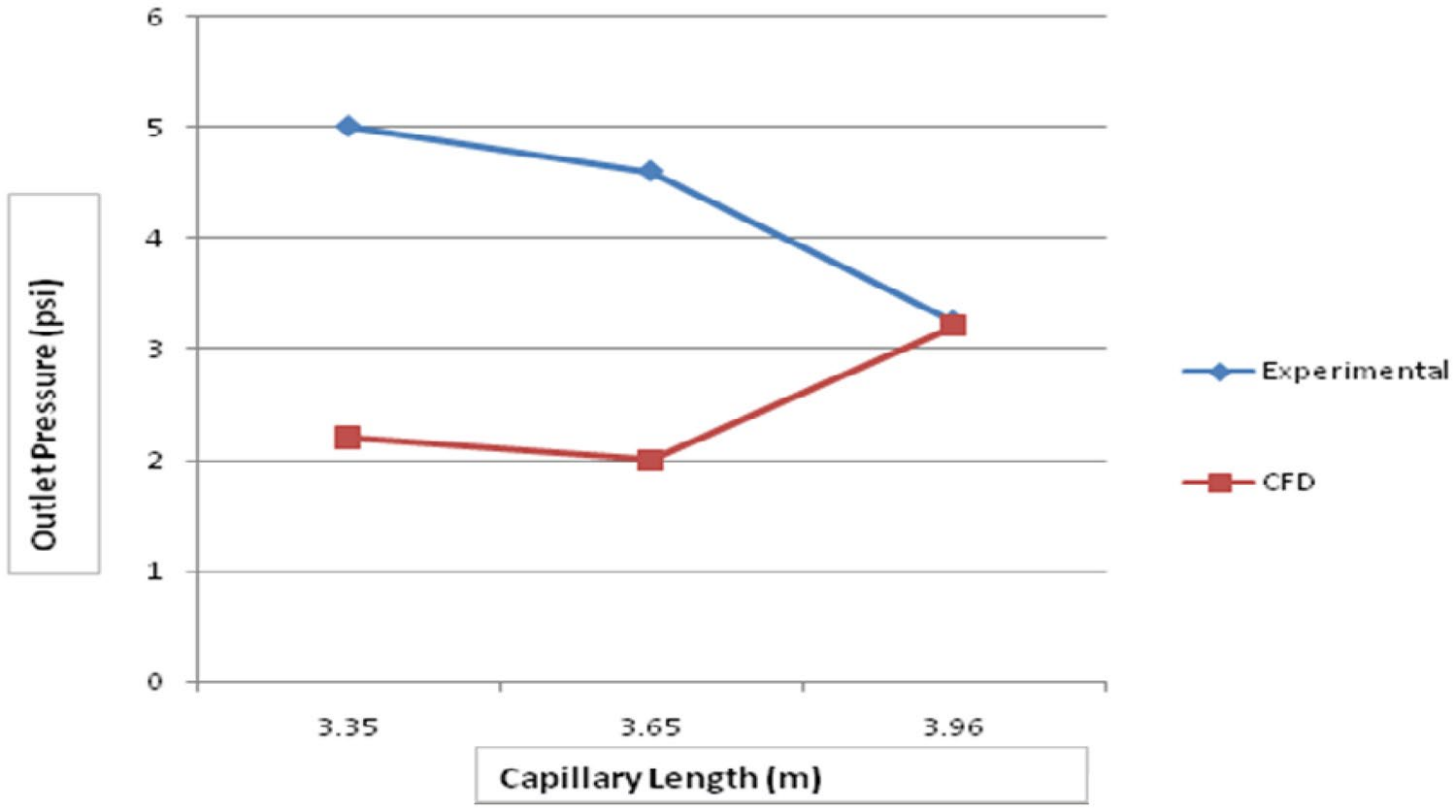
Variation of capillary outlet pressure with various tube lengths for R152a.

For validating the CFD results, existing experimental data are utilized. In this validation, the input parameters considered for experimental simulation were applied for CFD simulation. The obtained results are validated against the static pressure magnitudes. The obtained results indicate that the static pressure at the outlet of the capillary tube was less when compared to the inlet of the tube. The validation result shows that an increment in capillary tube length up to a certain limit decreases the pressure drop. Moreover, a decrement in static pressure drop between the inlet and outlet of the capillary tube increases the COP of the refrigeration system. The obtained CFD results agree well with the existing experimental results. The validation results are shown in Figs. 13, 14, 15 and 16. In this research, three different lengths of capillary tubes were utilized. The length of the tubes is 3.35 m, 3.65 m and 3.96 m. It was observed that the static pressure drop between the inlet and outlet of the capillary tube is increased when the tube length becomes 3.35 m. moreover, it was noted that the outlet pressure increases in the capillary tube when the tube size exists at 3.35 m.

Moreover, when the tube size is increased from 3.35 to 3.65 m, the pressure drop is decreased between the capillary tube’s inlet and outlet. It was observed that the outlet pressure of the capillary tube decreased drastically at the outlet. Due to this reason, the COP is increased at this length of the capillary tube. Further, an increment in the length of the tube from 3.65 to 3.96 m decreases the pressure drop again. It was observed that the pressure drop decreased below the optimum level at this length. It decreases the COP of the refrigerator. Therefore, the static pressure contours indicate that the 3.65 m length of the capillary tube produces optimum performance in the refrigerator. Moreover, an increment in the pressure drop increases the energy consumption.

## Conclusions

From the experimental findings, it is clear that refrigerant R152a has a lower refrigerating capacity as the length of the tube grows longer. The refrigeration capacity is maximum for the first coil (− 12 °C) and minimum for the third coil (− 16 °C). Maximum COP is obtained at an evaporator temperature of − 12 °C and capillary length of 3.65 m. The compressor power diminishes as the length of the capillary tube grows. The compressor power input is maximum at the evaporator temperature of − 12 °C and lowest at − 16 °C. The CFD and outlet pressure readings for capillary length are compared. Thus, it is seen that the circumstances are the same in both instances. The result is drawn that the system output enhances when the capillary length is improved to 3.65 m when compared to 3.35 m and 3.96 m. As a result, as the capillary length increases up to a specific amount, the system's performance improves.

Constraints necessitate the development of faster, simpler, and less expensive CFD techniques, even though the application of CFD in thermal-based industries and power plants will improve our comprehension of the dynamics and physics of a thermal analysis operation. This will help us optimize and design existing equipment. The advancement of CFD software will make automatic design and optimization a reality, and the creation of a web-based CFD will increase accessibility to the technology. All of these advancements will help CFD develop into an established field and potent engineering tool. The use of CFD in thermal engineering will therefore become more widely and quickly adopted in the future.

## Data Availability

The data are included within the article.
